# Transcriptomic Landscape of Paclitaxel-Induced Multidrug Resistance in 3D Cultures of Colon Cancer Cell Line DLD1

**DOI:** 10.3390/ijms26146580

**Published:** 2025-07-09

**Authors:** Sandra Dragicevic, Jelena Dinic, Milena Ugrin, Marija Vidovic, Tamara Babic, Aleksandra Nikolic

**Affiliations:** 1 Gene Regulation in Cancer Group, Institute of Molecular Genetics and Genetic Engineering, University of Belgrade, 11042 Belgrade, Serbia; tamara.babic@imgge.bg.ac.rs (T.B.); aleksandra.nikolic@imgge.bg.ac.rs (A.N.); 2 Department of Neurobiology, Institute for Biological Research “Siniša Stanković”—National Institute of the Republic of Serbia, University of Belgrade, 11108 Belgrade, Serbia; jelena.dinic@ibiss.bg.ac.rs; 3 Rare Disease Research and Therapeutics Development Group, Institute of Molecular Genetics and Genetic Engineering, University of Belgrade, 11042 Belgrade, Serbia; milena.ugrin@imgge.bg.ac.rs; 4 Plant Molecular Biology Group, Institute of Molecular Genetics and Genetic Engineering, University of Belgrade, 11042 Belgrade, Serbia; marija.vidovic@imgge.bg.ac.rs

**Keywords:** colon cancer, molecular mechanism, multidrug resistance, spheroid, transcriptomics

## Abstract

Multidrug resistance (MDR) significantly contributes to colon cancer recurrence, making it essential to understand its molecular basis for improved therapies. This study aimed to identify genes and pathways involved in resistance to standard chemotherapeutics by comparing transcriptome profiles of sensitive and paclitaxel-induced MDR colonospheres. Cell viability and growth were assessed following treatment with 5-fluorouracil, oxaliplatin, irinotecan, bevacizumab, and cetuximab. Drug concentrations in culture media posttreatment were measured using high-performance liquid chromatography (HPLC). RNA sequencing (RNA-seq) of untreated sensitive and resistant colonospheres identified differentially expressed genes linked to baseline resistance. Our results confirmed cross-resistance in the resistant model, showing highest oxaliplatin tolerance may involve mechanisms beyond efflux. Transcriptome analysis highlighted upregulation of *PIGR* and activation of the ribosomal signaling pathway as potential resistance mediators. Notably, *AKR1B10*, a gene linked to chemotherapeutic detoxification, was overexpressed, whereas genes related to adhesion and membrane transport were downregulated. The overexpression of ribosomal protein genes suggests ribosome biogenesis plays a key role in acquired resistance. These findings suggest that targeting ribosome biogenesis and specific deregulated genes such as *PIGR* and *AKR1B10* may offer promising strategies to overcome MDR in colon cancer.

## 1. Introduction

Chemoresistance is one of the major obstacles in the treatment of advanced colon cancer, and overcoming resistance remains one of the major challenges in colon cancer research. There is still an unmet need to better understand the underlying mechanism of chemoresistance in colon cancer as a complex phenomenon in order to achieve more promising clinical outcomes.

The first-line treatment option for patients with advanced colon cancer is the combination of chemotherapeutic agents specified in clinical practice guidelines [1]. Despite advances in treatment modalities over the past decade, the 5-year survival rate is still poor, and the main reason for treatment failure is the development of acquired resistance to all conventional chemotherapeutic agents, which occurs in 90% of patients with metastatic cancer [2]. The choice of drugs depends on the assessment of the treatment goals, the mutational profile of the tumor and, the toxicity profiles of the compounded drugs. Fluorouracil and folinic acid are considered the backbone of chemotherapy for colon cancer patients [3]. Oxaliplatin and irinotecan, both commonly used in standard treatment regimens, have distinct toxicity profiles but demonstrate similar efficacy [4,5,6]. Three biological agents (bevacizumab, cetuximab, and panitumumab) are also approved for the first-line treatment of metastatic colon cancer [7]. Bevacizumab is most commonly used, as it is the only biological agent that can be used in combination with cytotoxic chemotherapy for tumors with mutated *RAS* [8].

Reproducing chemoresistance in the laboratory setting provides a better insight into the mechanisms underlying this complex process. In addition to resistance to single drugs, multidrug resistance (MDR) models have also been developed [9,10,11,12]. Systematic approaches are needed to better understand the key altered processes in cells that are resistant to chemotherapy, especially when it comes to MDR. Large-scale expression analysis of cell models is an attractive strategy to study MDR, as it is a multifactorial phenomenon involving multiple genes and pathways. It can result from a variety of cellular mechanisms, such as activation or overexpression of drug export proteins, alteration of target enzymes, increased drug metabolism and elimination due to altered expression of cytochrome P450 enzymes, changes in cell cycle control or altered apoptotic threshold, increased intracellular drug inactivation due to conjugation with glutathione, and altered DNA repair capacity [13,14]. Therefore, high-throughput analyzes of MDR cell models can reveal the underlying mechanisms and point to genes and pathways that can be targeted for novel cancer therapies.

The DLD1 cell line, which represents an advanced stage of colon cancer (stage III, Dukes’ C), is widely used to study colorectal cancer (CRC) progression and to evaluate the efficacy of anticancer agents. DLD1 cells are microsatellite stable and harbor mutations in genes *APC*, *KRAS*, *TP53*, and *PIK3CA*. Mutations in *KRAS* and *TP53* are found in approximately 40% and up to 60% of CRCs, respectively, while *APC* mutations occur in over 70% of cases and *PIK3CA* in about 15–20% [15,16,17]. These mutations are frequently observed in advanced stage tumors (stage III–IV), and at least one of them is present in 58.9% of patients with stage III disease [18]. Moreover, the co-occurrence of *APC*, *KRAS*, and *TP53* mutations has been reported in approximately 32% of CRC cases in The Cancer Genome Atlas (TCGA) cohort [19]. Considering that advanced-stage adenocarcinomas account for a substantial proportion of newly diagnosed CRCs—approximately 61% in individuals under 50 and 47% in those over 50 years—DLD1 cells provide a clinically relevant model for studying resistance mechanisms in a large subset of patients [20].

In the cell model used for this study, the DLD1-TxR cell line, the MDR phenotype was previously developed by continuous exposure of the DLD1 cell line to gradually increasing concentrations of paclitaxel, while the phenotype was confirmed by cross-resistance to five other anticancer drugs (vinblastine, doxorubicin, cisplatin, etoposide, and epirubicin) [21]. The DLD1-TxR cell line is characterized by overexpression of the *ABCB1* gene and downexpression of the *ABCC1* gene, which encode the membrane transporters P-glycoprotein (P-gp) and MDR-associated protein 1 (MRP1), respectively [21]. P-gp, which contributes to chemoresistance through drug export from cancer cells, is also overexpressed [22]. The karyotypic features of the MDR cell line are similar to those of its sensitive counterpart, but it acquired some novel chromosomal aberrations, with a loss of genomic material observed at chromosomes 6q and Y [22]. This cell line has demonstrated reliability as MDR model system for testing various natural and synthetic compounds with potential anti-cancer activity [23,24,25,26,27,28,29,30,31,32].

Three-dimensional (3D) cell cultures such as spheroids represent a more advanced model compared to conventional two-dimensional (2D) cell cultures, as they better mimic the natural microenvironment of tumor cells. They allow a more realistic study of the interactions of cells with their environment, which is crucial for understanding phenomena such as MDR. These cultures allow for a more accurate assessment of cancer drug efficacy and resistance mechanisms [33,34]. Moreover, since 3D models better mimic in vivo conditions, their transcriptome differs significantly from that of 2D models [35,36].

The aim of this study was to identify genes and pathways involved in resistance to conventional chemotherapeutic agents for the treatment of colon cancer by analyzing transcriptome profiles of sensitive and paclitaxel-induced MDR colonospheres.

## 2. Results

### 2.1. Influence of the Therapeutic Agents on Cell Viability and Growth

The sensitive cell line DLD1 and the MDR cell line DLD1-TxR grown in 2D were treated with conventional chemotherapeutics (5-fluorouracil, oxaliplatin, and irinotecan) and biological agents (bevacizumab and cetuximab). To determine the half-maximal inhibitory concentration (IC_50_) and calculate the relative resistance factor (Rf) for each therapeutic agent, the viability of the adherent cells after treatment was assessed using the 3-[4,5-dimethylthiazol-2-yl]-2,5-diphenyltetrazolium bromide (MTT) assay. In addition to the previously published Rf for paclitaxel, the IC_50_ values and Rfs determined in the current study are shown in Table 1. Among the conventional chemotherapeutic agents, an extremely high Rf was determined for oxaliplatin (Rf = 1040) (Supplementary Figure S1). DLD1-TxR cells showed moderate resistance to 5-fluorouracil (Rf = 3.8) and minimal resistance to irinotecan (Rf = 1.4). Neither the sensitive DLD1 nor the resistant cell line DLD1-TxR responded to treatment with biological agents.

**Figure 1:**
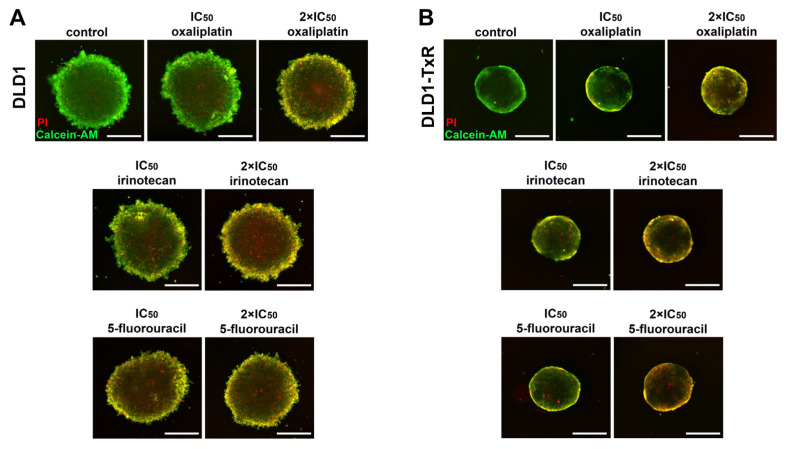
Cell viability of spheroids treated with oxaliplatin, irinotecan, and 5-fluorouracil. The viability of sensitive (**A**) and resistant **(B**) spheroids after 72 h of treatment with oxaliplatin, irinotecan, and 5-fluorouracil was visualized by fluorescence microscopy. The representative images show living cells stained with calcein-AM (green) and dead cells stained with propidium iodide (PI) (red). Control images correspond to untreated spheroids at the experimental endpoint. Experiments were repeated at least three times (n ≥ 3). Scale bar = 350 µm. IC_50_, the half-maximal inhibitory concentration.

Based on MTT assay results, spheroids were subsequently treated with conventional chemotherapeutic agents at equi-effective doses, corresponding to each cell line IC_50_ and 2 × IC_50_ values. Since 3D cultures are generally less sensitive to treatment than 2D models, this approach enabled a more biologically relevant evaluation of colonosphere response. Fluorescence microscopy was used to visualize changes in spheroid viability and growth following treatment, with representative images presented in Figure 1, illustrating the effects of both low (IC_50_) and high (2 × IC_50_) drug concentrations.

**Figure 2:**
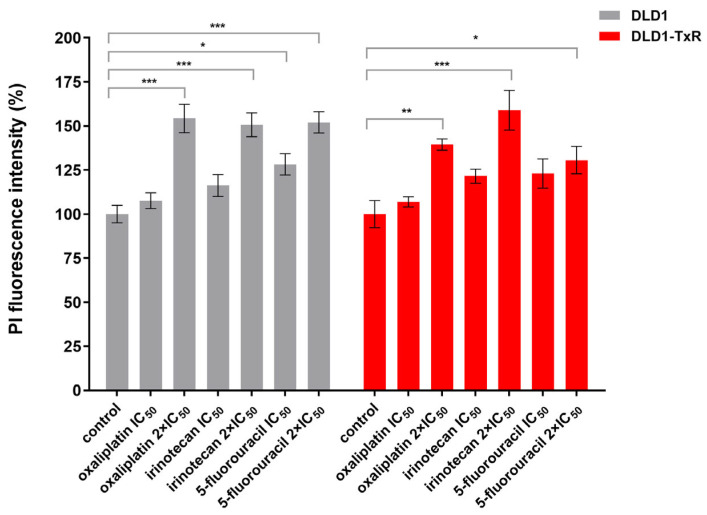
Quantification of cell viability of spheroids after treatment with oxaliplatin, irinotecan, and 5-fluorouracil. The histogram shows the quantification of PI-labeled (dead) cells in sensitive and resistant spheroids after 72 h of treatment with oxaliplatin, irinotecan, and 5-fluorouracil. Experiments were performed at least three times (n ≥ 3), and data are presented as mean ± SEM. Statistically significant differences between treated and control groups were determined by one-way ANOVA followed by Dunnett’s multiple comparisons test and are marked with * (*p* < 0.05), ** (*p* < 0.01), and *** (*p* < 0.001). No statistically significant differences were observed between DLD1 and DLD1-TxR spheroids for any treatment condition (Tukey’s multiple comparisons test, *p* > 0.47; Supplementary Table S1). IC_50_, the half-maximal inhibitory concentration; PI, propidium iodide.

Statistical analysis showed that all chemotherapeutic agents used at higher concentrations significantly reduced the viability of the spheroids (*p* < 0.005), as shown by propidium iodide (PI) staining, indicating an increase in dead cells. At lower concentrations, a similar trend was detected; however, statistical significance was found only for sensitive spheroids in response to 5-fluorouracil (*p* = 0.013) (Figure 2).

Statistical comparison of corresponding drug treatments (IC_50_ and 2 × IC_50_) between DLD1 and DLD1-TxR spheroids revealed no significant differences for oxaliplatin, irinotecan, or 5-fluorouracil (Tukey’s multiple comparisons test, *p* > 0.47 for all comparisons; Supplementary Table S1).

**Figure 3:**
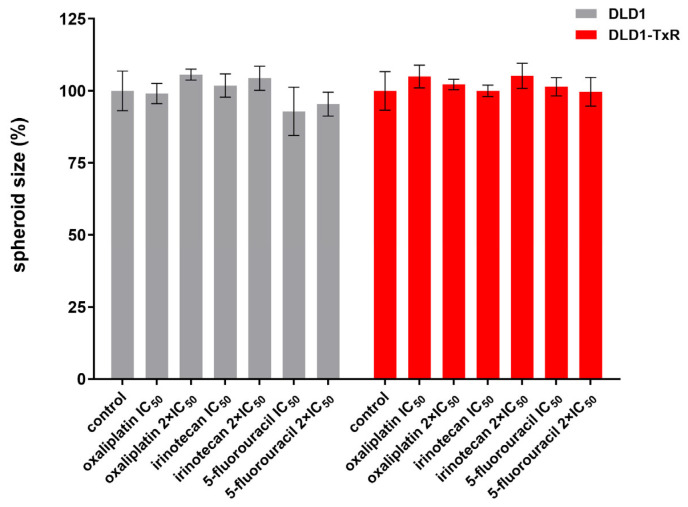
Quantification of spheroid size after treatment with oxaliplatin, irinotecan, and 5-fluorouracil. The histogram shows the quantification of spheroid size for both sensitive and resistant spheroids after 72 h of treatment with oxaliplatin, irinotecan, and 5-fluorouracil. Experiments were repeated at least three times (n ≥ 3), and data are expressed as mean ± SEM. No statistically significant differences were observed between groups (one-way ANOVA, *p* > 0.05). IC_50_, the half-maximal inhibitory concentration.

We observed no changes in the size of spheroids in response to any of the treatments (*p* > 0.05) (Figure 3).

### 2.2. Analysis of the Drug Efflux

To analyze drug efflux, sensitive and resistant cells were treated with conventional chemotherapeutics using the IC_50_ concentrations determined for each cell line. The presence of the applied drugs in the medium in which the cells were grown after treatment was analyzed by high-performance liquid chromatography (HPLC), and none of the chemotherapeutic agents were detected according to the established limit of detection (LOD).

### 2.3. Genomic and Transcriptomic Profiling

Whole-exome sequencing (WES) analysis was performed on DNA isolated from both the sensitive and resistant spheroids. The results showed the loss of the following sequence variants characteristic of the DLD1 cell line in the sensitive phenotype, while they were retained in the drug-resistant phenotype: *KRAS* G13D, *PIK3CA* E545K, *PIK3CA* E549N, *ERBB3* N126K, *TP53* S241F, *IDH1* G97D, and *KDR* S925F. Changes in other oncogenes and tumor suppressors, which are frequently deregulated in colon cancer, were not detected in the spheroids analyzed.

**Figure 4:**
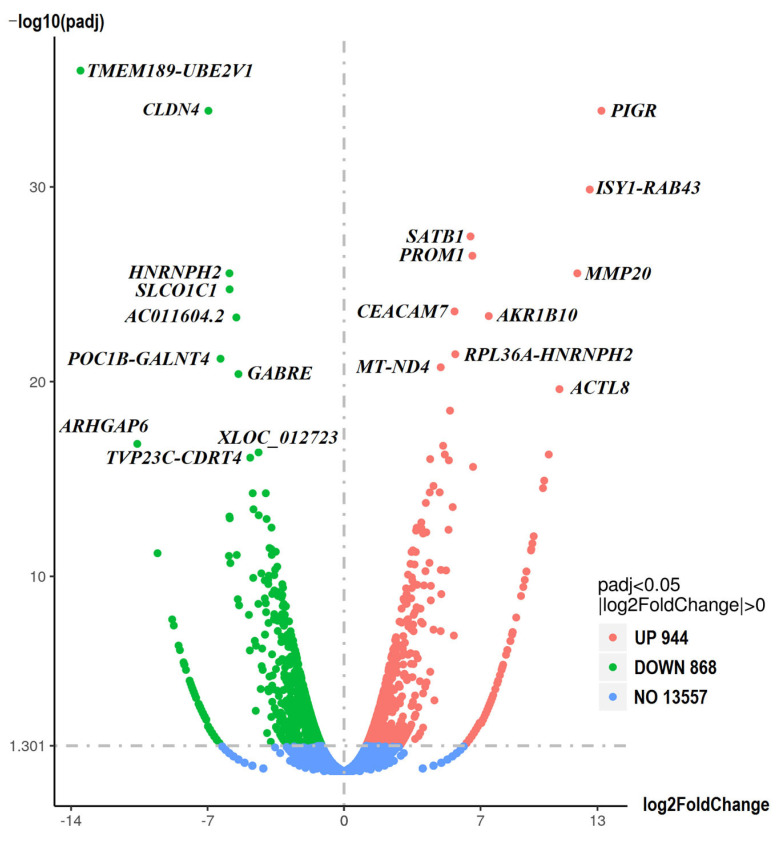
Differentially expressed genes (DEGs). The volcano plots illustrate the magnitude and significance of DEGs between sensitive and resistant spheroids. The *Y*-axis (scaled as −log10 (*p*-value)) represents the statistical significance of the change in gene expression, while the *X*-axis (scaled as log2 fold change) shows the magnitude of the change in gene expression. Significantly upregulated and downregulated genes are highlighted in red and green, respectively. Genes whose expression does not differ between the two samples are highlighted in blue.

RNA sequencing (RNA-seq) analysis was performed to determine the differentially expressed genes (DEGs) between sensitive and resistant cells. The results are shown in Figure 4. Based on the fold change and significance level, the most upregulated and downregulated genes in the resistant spheroids are shown in Supplementary Table S2 and Table S3, respectively.

To further investigate the expression of genes identified as the most significantly altered in our study, we compared our results with publicly available transcriptomic datasets (GSE196900 and GSE138647), including HCT116 and SW480 chemoresistant colon cancer cell lines. *AKR1B10* gene expression was increased in HCT116 cells resistant to 5-fluorouracil, whereas an opposite trend was observed in SW480 cells under similar treatment conditions. Regarding oxaliplatin resistance, HCT116 cells exhibited reduced expression of genes *AKR1B10* and *PIGR*.

While not ranked among the topmost DEGs, *ABCB1*, which encodes a membrane transporter P-gp involved in drug efflux, was significantly overexpressed in the resistant DLD1-TxR spheroids compared to the sensitive parental line (log2FoldChange = 3.46, padj = 5.6 × 10^−12^). In contrast, *ABCC1* expression encoding MRP1 remained low and was not significantly altered in resistant spheroids. Expression of *SLC22A2*, encoding organic cation transporter 2 (OCT2), remained unchanged between resistant and sensitive spheroids.

**Figure 5:**
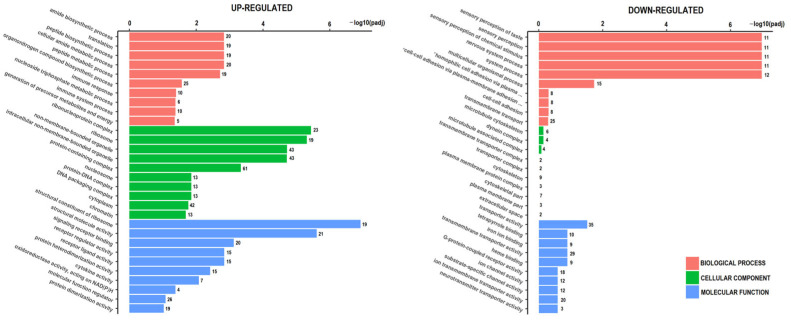
Gene ontology (GO) enrichment analysis for upregulated and downregulated genes between sensitive and resistant spheroids. The *Y*-axis shows the pathway terms (biological process, cellular component, and molecular function), while the *X*-axis (scaled as −log10 (*p*-value)) represents the statistical significance of the enrichment results. * plasma-membrane adhesion molecules.

Gene ontology (GO) enrichment analysis of DEGs was performed to identify biological functions that are overrepresented in resistant spheroids (Figure 5). The most enriched biological pathways in the list of upregulated DEGs were ribosome-related, while the biological processes that were particularly abundant in the downregulated genes were related to sensory perception, including sensory perception of chemical stimuli.

## 3. Discussion

In the present study, we investigated the molecular basis of paclitaxel-induced MDR in a colon cancer spheroid model. We evaluated the genomic and transcriptomic profiles of the sensitive DLD1 cell line and the derived resistant DLD1-TxR cell line to identify genes and pathways that could potentially be targeted to overcome resistance.

To our best knowledge, the DLD1-TxR cell line is the only MDR colon cancer cell model established using selective pressure of paclitaxel. Its mechanism of action differs from 5-fluorouracil, irinotecan, and oxaliplatin, which act directly or indirectly at the DNA level. Paclitaxel has an antimitotic effect by stabilizing microtubules, leading to cell cycle arrest and inhibition of cell proliferation. Given that paclitaxel is not routinely used in colon cancer treatment and that the DLD1-TxR cell model was previously established, we employed this model in the present study as a tool to investigate molecular pathways underlying resistance to standard chemotherapeutics used in colon cancer. By employing an MDR colon cancer cell model in which resistance was induced by an agent not commonly used in colon cancer therapy, we were able to explore molecular adaptations that were not driven by direct exposure to standard chemotherapeutics yet conferred broad resistance to them—offering insights into pre-existing mechanisms that may enable colon cancer cells to evade conventional treatment.

First, we performed treatment under 2D culture conditions in order to determine the IC_50_ values for all therapeutics and to calculate Rf of the DLD1-TxR cells. Growing cells as a monolayer allows equal exposure of each cell to the therapeutics, which is critical for accurate IC_50_ determination. This approach is consistent with our previously established methodology and was used to ensure comparability across studies as well as to define appropriate concentrations for subsequent experiments. In line with previously published data showing that the DLD1-TxR cell model exhibits stable cross-resistance to various therapeutics, our current study also demonstrated cross-resistance of DLD1-TxR cells to the most commonly used chemotherapeutic agents in colon cancer [28,30,31,32]. A significantly higher Rf was recorded for oxaliplatin compared to 5-fluorouracil and irinotecan. In a monolayer setting, oxaliplatin might exhibit a more pronounced cytotoxic effect due to its ability to induce DNA cross-links, a mechanism that leads to cell death independently of cell cycle progression. Oxaliplatin can exert its effect even in slowly proliferating cells, which may contribute to its higher potency in vitro. In contrast, 5-fluorouracil and irinotecan do not act directly on DNA and rely on active cell proliferation. Cetuximab and bevacizumab showed no significant cytotoxic effect. This result might be expected, given that biological drugs target specific signaling pathways or extracellular molecules rather than inducing direct cytotoxicity in vitro. Bevacizumab targets vascular endothelial growth factor and exerts its therapeutic effect primarily through inhibition of angiogenesis, a mechanism not replicable in our in vitro setting due to the absence of vascular components [8]. On the other hand, cetuximab acts via inhibition of epidermal growth factor receptor (EGFR). However, DLD1 cells harbor a *KRAS* mutation, which is a known predictor of resistance to anti-EGFR therapy [37]. Our study showed no difference in *EGFR* gene expression between the sensitive and resistant phenotypes, confirming the overall low expression of the receptor. Taking these factors into account, along with the lack of direct cytotoxicity observed in 2D, biological agents were suggested as ineffective in the 3D model and were not further tested in this context.

Since 3D models generally exhibit reduced sensitivity to therapy compared to 2D monolayer cultures, the effects of conventional chemotherapeutics on cell growth and viability were further investigated using spheroid cultures. Culturing cells as spheroids mimics the architecture of tumor tissue and better reflects the molecular characteristics of sensitive and resistant cells as found in vivo [33]. Considering the compact structure of spheroids, enhanced cell–cell interactions, and limited drug penetration, we applied equi-effective dosing—using cell line-specific IC_50_ and 2 × IC_50_ concentrations—to ensure biologically comparable levels of drug exposure and to better reflect the differential response between 2D and 3D cultures. Our results showed that none of the chemotherapeutic agents used had any effect on spheroid growth, suggesting that their effects on proliferation were minimal. In contrast, all chemotherapeutic agents at high concentrations significantly impaired the viability of both sensitive and resistant cells. At lower concentrations, resistant spheroids maintained resistance to all agents, whereas a significant effect was observed only for 5-fluorouracil in sensitive spheroids. While differences in spheroid viability between sensitive and resistant cells at equi-effective doses were modest, this does not negate the presence of resistance. Rather, it indicates that resistant spheroids require higher drug concentrations to achieve similar biological effects, highlighting their reduced sensitivity. These findings confirm that the compactness of spheroids plays a significant role in modulating treatment response. The obtained result may be explained by the fact that 5-fluorouracil targets rapidly proliferating cells, which are typically located at the outer layers of spheroids. Additionally, 5-fluorouracil may penetrate spheroids more efficiently, as it is a small hydrophilic molecule compared to irinotecan and oxaliplatin, which are larger and more hydrophobic. When compared to clinically achievable plasma concentrations, the IC_50_ and 2 × IC_50_ doses of irinotecan in both sensitive and resistant cells were close to the maximum reported value (5.78 µM) [38]. For 5-fluorouracil, the IC_50_ and 2 × IC_50_ doses used in both spheroid phenotypes were below the reported plasma concentration range (160–426 µM) [39,40,41]. Similarly, the IC_50_ dose of oxaliplatin used in sensitive spheroids was below the clinically relevant range (3–12 µM). In contrast, both the IC_50_ and 2 × IC_50_ concentrations of oxaliplatin used for treatment greatly exceeded the clinically achievable range, indicating pronounced resistance [42,43,44]. The phenomenon that the concentrations needed to inhibit the growth of resistant cells often exceed clinically achievable plasma levels highlights a common feature of resistance models and reflects a high degree of cellular adaptation to the drug action. Exploring a wider range of drug concentrations (IC_10_–IC_90_) through comprehensive dose–response analyses could provide additional valuable functional insights into spheroid response to treatment. Although the in vitro concentrations used may not fully reflect clinical applicability, they nonetheless provide a useful basis for further characterization of resistance features in a controlled experimental setting. The lack of effect at physiologically relevant concentrations may indicate a combination of intrinsic cellular resistance mechanisms and additional protection conferred by the 3D structure.

Cross-resistance of DLD1-TxR cells to the P-gp substrates vinblastine, etoposide, doxorubicin, and epirubicin has already been demonstrated, with Rf values ranging from 14-fold to 28-fold [21]. Although P-gp has been shown to play an important role in the transport of irinotecan out of the cancer cell, its involvement in resistance to 5-fluorouracil and platinum-based drugs has also been reported [45]. The Rf for cisplatin previously determined for DLD1-TxR cells was 3.2-fold [21]. Knowing that P-gp is overexpressed in DLD1-TxR cells, we investigated drug efflux by measuring drug transport from inside the cells to the extracellular medium as a potential underlying MDR mechanism. Since drug efflux mechanisms are largely governed by intrinsic cellular transporters, which function similarly regardless of culture dimensionality, we performed these assays in 2D cultures to ensure uniform drug exposure and accurate quantification of efflux activity. Our RNA-seq data revealed significant upregulation of the *ABCB1* gene in resistant DLD1-TxR spheroids, consistent with its well-established role in mediating drug efflux and chemoresistance [21]. This finding suggests that P-gp-mediated efflux contributes to the resistant phenotype, which is supported by our previous observations in 2D cultures, where DLD1-TxR cells showed increased rhodamine 123 efflux that was reversible with verapamil, indicating functional P-gp activity [31]. Notably, *ABCC1* expression remained low and was not significantly altered in resistant spheroids, implying that among the evaluated efflux transporters, P-gp may play a more relevant role. The absence of detectable chemotherapeutic drugs in the post-treatment medium does not rule out the role of membrane transporters in drug efflux, but it also indicates that additional, alternative resistance mechanisms might be involved. These could include intracellular drug metabolism, sequestration, or altered drug targets, as suggested by prior studies on 5-fluorouracil resistance and enzyme variability across cell lines [46]. Of note, oxaliplatin is not typically considered a substrate for P-gp, and the pronounced resistance to this agent observed in DLD1-TxR spheroids is unlikely to be solely explained by *ABCB1* upregulation. Expression of *SLC22A2*, encoding the principal uptake transporter for oxaliplatin (OCT2), remained unchanged between resistant and sensitive spheroids, suggesting that oxaliplatin uptake is likely similar in both cell types. Taken together, these findings indicate that mechanisms beyond drug transporters might be involved in the MDR phenotype observed in 3D culture. Further functional studies in 3D cultures are necessary to clarify the role of drug transporters, especially P-gp, and to identify other mechanisms underlying the observed resistance phenotype.

Since the lack of cellular response to conventional chemotherapeutic agents may be related to the deregulation of various genes, we analyzed both the sensitive and MDR cell phenotypes for the presence of genetic alterations. In order to match the conditions used for transcriptomic analysis, WES analysis was carried out on DNA isolated from 3D spheroid cultures, although the culture system was not expected to impact the genetic variant profiles. All variants characteristic of the parental DLD1 cell line were detected in the resistant phenotype, whereas they were lost in the sensitive phenotype. The retention of DLD1-characteristic genetic variants in resistant phenotype, compared to their loss in the sensitive cell line, highlights the dynamic nature of cancer cell genomes. Similar observations in DLD1 cells were previously reported, demonstrating that genetic instability, combined with selective pressures such as drug exposure or prolonged culture, contributes to the retention or loss of specific alterations [47,48]. Both cell lines, namely DLD1 and DLD1-TxR, have been extensively utilized over many years in our previous studies. In the sensitive phenotype, the absence of external stimuli may lead to genetic drift or reversion, whereas the resistant phenotype retains key genetic alterations to maintain a survival advantage under chemotherapeutic stress. None of the cell lines acquired variants typical of other colon cancer oncogenes or tumor suppressors that could give them a selective advantage in the presence of chemotherapeutic agents. This suggests that dysregulation of gene expression is more important for resistance mechanisms.

Although the phenotypic differences in drug response were more prominent in 2D cultures, resistance was also observed in spheroids at the same concentrations used. Since spheroids better reflect the structure and intracellular interactions of the tumor in vivo, transcriptome profiling in this model provides more physiologically relevant insights. To gain a deeper understanding of the molecular basis of the observed phenotype, we analyzed the basal gene expression landscape of untreated spheroids, aiming to capture transcriptomic alterations not influenced by acute drug exposure. This approach enabled us to identify genes and pathways potentially contributing to the MDR state, offering a broader view of resistance mechanisms. Few genes showed a similar dysregulation pattern when compared with results of recent studies based on the analysis of gene expression profiles of cell lines resistant and non-resistant to oxaliplatin [49,50]. The differences in gene expression signatures could be due to a variety of factors: cell models, agents used to generate resistance, doses applied, and/or the time frame in which resistance was generated. On the other hand, some previously established mechanisms of resistance to oxaliplatin have been observed, such as the increased expression of genes *ALDH1L1* and *POLA* [51,52].

Among the upregulated genes in resistant spheroids, the *PIGR* gene was overexpressed with the highest statistical significance. This finding is consistent with reports in pancreatic cancer, where it has also been associated with chemoresistance [53]. It has been suggested that the polymeric immunoglobulin receptor encoded by the *PIGR* gene stimulates activation of the ribosomal pathway in hepatocellular carcinoma [54]. Contribution of this transmembrane protein to chemoresistance through the epithelial–mesenchymal pathway was demonstrated. Colon cancer cells RKO and HCT8 overexpressing PIGR protein were shown sensitive to cisplatin but not to oxaliplatin [55]. In our study, we found that overexpression of the *PIGR* gene in combination with ribosomal pathway activation identifies this gene and its protein product as potential targets to overcome resistance. Further investigation of the *PIGR* gene, which has already been studied as a diagnostic and prognostic marker in various cancers of uncertain outcome, should be shifted to advanced tumors and the context of adjuvant therapy [56].

The finding that the *AKR1B10* gene is one of the genes overexpressed in resistant spheroids with high statistical significance is not surprising. This gene is primarily expressed in gastrointestinal tissues and encodes the aldo/keto reductase family member 1B10, which plays a role in reducing oxidative stress induced by environmental stimuli [57]. This finding also supports one of the previously proposed mechanisms of drug resistance mediated by aldo reductases involving the elimination of cytotoxic metabolites [58]. For this reason, a protective role of the highly expressed *AKR1B10* gene in MDR cells could be suggested, assuming that these cells possess an initially elevated capacity for defense against oxidative stress due to this high basal expression. Although the *AKR1B10* gene has the potential to be considered a valuable therapeutic target, the mechanisms underlying the regulation of the *AKR1B10* gene are complex and unexplored regarding chemoresistance. It has been shown that *AKR1B10* gene expression can change in the opposite direction in response to therapeutic pressure, depending on the cancer cell type and anticancer agents applied as well as the mutational status of p53 [59]. In the colon cancer cell line HCT116 with wild-type p53, treatment with oxaliplatin induced *AKR1B10* gene expression significantly more than other chemotherapeutic agents, while in the colon cancer cell line HT29 with mutated p53, it had a significant effect but in the opposite direction [60]. The DLD1-TxR cells in our study carry a mutated *TP53* gene, which could affect how *AKR1B10* gene expression responds to oxaliplatin treatment. It can be assumed that the initially high basal expression of the *AKR1B10* gene provides sufficient protection against oxidative stress, thereby maintaining resistance even if its expression might be partially downregulated upon treatment.

To further explore the relevance of selected genes identified in our transcriptomic analysis, we compared our findings with publicly available datasets of colon cancer cell lines with acquired resistance to 5-fluorouracil and oxaliplatin, including HCT116 and SW480 models. Expression of these genes varied depending on the cell line and resistance model, with the *AKR1B10* gene showing a relatively consistent association with resistance, while *PIGR* gene expression was more variable. Interestingly, in our model, DLD1-TxR cells with high expression of both genes showed particularly poor response to oxaliplatin, suggesting a possible role for these genes in MDR as well. This highlights the cell line- and drug-specific nature of resistance mechanisms, complicating direct extrapolation across models. Nonetheless, the observed patterns suggest a potential involvement of these genes in MDR, warranting further investigation to clarify their roles in different colon cancer models.

The results of our study support previous findings that cancer cells acquire characteristics similar to cancer stem cells to achieve resistance to chemotherapeutics. In addition to the upregulation of the *PROM1* gene, which encodes the common marker for cancer stem cells CD133, the *ISY1-RAB43* gene, proposed as a novel marker for cancer stem cells, was also upregulated [61]. A link between overexpressed CD133 and high chemotherapy resistance has been observed previously, and an in vitro study showed that colon cancer cells can switch from CD133- to CD133+ in response to environmental stimuli [62]. Transcriptomic changes in MDR cells involve not only genes that allow an individual cell to become more resistant to environmental stimuli but also genes associated with modulation of the tumor microenvironment. Among the matrix metalloproteinase genes, the *MMP20* gene was overexpressed in MDR spheroids. A previously performed multi-omics analysis showed the variability of *MMP20* gene expression in different databases when colon cancer tissue was compared with normal gut mucosa [63]. Based on these results, it could be hypothesized that increased expression of the *MMP20* gene, in contrast to other *MMP* genes, is associated with chemoresistance rather than colon cancer progression.

GO analysis of our study revealed that most of the upregulated genes in resistant spheroids are associated with ribosomal processes. This finding is consistent with the results of a previous proteomics study, which showed that ribosomal processes were upregulated in the DLD1 cell line with established chemoresistance by continuous treatment with a 5-fluorouracil/oxaliplatin combination [64]. The Mammalian Target of Rapamycin (mTOR) signaling pathway plays a key role in the coordination of all components required for ribosome synthesis [65]. The high activity of this signaling pathway in cancer cells contributes to their drug resistance [66]. Our results support these findings, as the expression of the gene *EIF4EBP1*, which encodes a downstream effector of the mTOR signaling pathway, was also increased in resistant spheroids.

We found that the *TMEM189-UBE2V1* transcript was most strongly downregulated in the resistant spheroids. The *TMEM189* and *UBE2V1* genes are neighboring genes that are normally expressed separately and are thought to be targets in different cancer types [35,67,68]. However, they may also be naturally transcribed but rarely as a *TMEM189-UBE2V1* fusion transcript with unknown function. Another significantly downregulated gene was *CLDN4*. The tight junction protein encoded by the *CLDN4* gene has been associated with gastric cancer progression and reduced sensitivity to chemotherapy [69]. In colon cancer, however, the results regarding protein expression levels are contradictory [69]. In addition to several genes encoding adhesion proteins (*CLN4*, *PCDHA9*, *DSG1*, *ITG2A2B*, and *GJA9*), genes encoding membrane transport proteins (members of the *SLC* gene families) and binding proteins (*CFH* and *FABP3*) were also downregulated. In this way, resistant cells could increase their migration potential and invasiveness as well as their resistance through reduced uptake of chemotherapeutic agents. Our finding that the downregulated genes in resistant spheroids are mainly related to the response to chemical stimuli might be the expected result.

The current study was designed as a preliminary mechanistic investigation to establish a foundation for future translational work. The genes discussed (such as *PIGR* and *AKR1B10*) were selected based on strong differential expression and known relevance to drug resistance. However, these findings require further validation across multiple resistant colon cancer cell lines and comprehensive analysis in clinical samples to fully assess their predictive potential. Information on the gene expression signature of resistant cancer cells could be crucial for developing molecular assays to predict response to conventional chemotherapy, particularly oxaliplatin-based therapy, as suggested by our results. The ability to predict tumor response would be of great importance in guiding alternative therapeutic approaches in patients classified as non-responders.

Transcriptome profiling is one of the most widely applied methods for exploring human diseases at the molecular level, and our study was focused on transcriptomic analysis of protein coding genes. It was shown that nuclear factor erythroid 2-related factor 2 is constantly active in response to stress and directly induces transcription of target genes including *AKR1B10*. Previous studies showed increase and decrease in the resistant phenotype of different cancer cells depending on overexpression or knockdown of this transcription factor. Activation of activator protein 1, a transcriptional stimulator, and the receptor tyrosine kinase/ERK cascade was also proposed as a significant mechanism involved in modulation of *AKR1B10* gene expression [59]. Besides transcriptional regulators, epigenetic changes could contribute to mediating drug resistance and should be taken into consideration. Expression of *PIGR* gene was shown to negatively correlate with promoter methylation and cancer prognosis [55]. However, in our study, this gene was found upregulated, suggesting other underlying mechanisms. In 5-fluorouracil-resistant colon cancer cells, it was shown that demethylation of *DUOX2* gene promoter, encoding dual oxidase 2, is associated with promotion of epithelial-mesenchymal transition and increased oxidative stress [70]. Additionally, non-coding RNA molecules play a crucial role in regulation of epigenetic processes by recruiting enzymes involved in methylation or histone acetylation and interacting with chromatin as well. They also regulate protein translation by influencing mRNA stability [71]. Some of these mechanisms might be suggested to contribute alterations in gene expression found in our study. Upregulation of drug-degrading enzymes, changes in apoptosis, and dysregulation of signaling pathways that control cell adhesion and the tumor microenvironment are frequently seen in colon cancer [72]. These changes are not only associated with altered expression of key genes and downstream proteins but may also involve modification in the structure and function of proteins through post-translational modifications, which can influence cancer cell characteristics and promote a resistant phenotype. Future research should address mechanisms of action of resistance-related genes and their upstream and downstream pathways by applying functional validation and pathway analysis.

The resistant DLD1-TxR cell line used in our study has been shown to express a large number of genes involved in ribosome biogenesis. Dysregulation of ribosomal proteins has already been shown to be important for cancer development and resistance to therapy [73,74]. During cancer therapy, cancer cells respond to the selective pressure of chemotherapeutic agents by increasing the synthesis of proteins that allow them to grow and survive. This adaptive response correlates with aggressive tumor behavior and leads to poor survival prognosis. Recent studies have highlighted the potential of using selective inhibitors of ribosome biogenesis as a promising therapeutic strategy [75,76,77]. Our results suggest that the burst in ribosome biogenesis is a common point in the development of MDR in cancer cells that develops independently of the type of drug that induces resistance. Based on these findings, we propose that co-targeting ribosome biogenesis and the mTOR signaling pathway may offer a novel therapeutic angle for addressing MDR in colon cancer. Such a strategy has the potential to improve treatment outcomes by attenuating protein synthesis and limiting tumor adaptability. In this context, the use of inhibitors of ribosomal protein synthesis, such as cycloheximide, in combination with chemotherapeutic agents could provide direct functional validation of this hypothesis in future studies. Nevertheless, these hypotheses require validation through comprehensive in vitro and in vivo studies to confirm their translational relevance.

The present study provides important insights into the role of ribosomal signaling and genes such as *PIGR* and *AKR1B10* in acquired MDR. The results suggest that ribosome biogenesis and associated processes may represent common mechanisms underlying resistance to different classes of drugs. However, some limitations should be taken into consideration. This study was conducted using only one pair of colon cancer cell lines: the parental DLD1 and its drug-resistant derivative DLD1-TxR. While this model allows for a controlled investigation of resistance-associated changes, it does not account for the broader heterogeneity of resistance mechanisms that may exist among different CRC models. Paclitaxel-induced resistance primarily involves alterations in the expression of multidrug efflux transporters, microtubule dynamics, and apoptosis [78]. It is known that colon cancer develops resistance through a variety of mechanisms beyond paclitaxel treatment. Future research with additional MDR models, including those induced by conventional chemotherapeutic agents for colon cancer, will be crucial to validate the relevance of our findings in a broader context. Additionally, 3D cell co-cultures, such as heterogeneous spheroids, could provide deeper insights into the complexity of MDR, including intercellular transfer of resistance. Extracellular vesicles are highlighted as essential players in the transport of bioactive compounds that can modify the characteristics and functions of sensitive tumor or stromal cells [79]. Elucidation of this aspect of MDR would significantly contribute to the development of strategies to overcome resistance and improve the effectiveness of cancer treatments.

## 4. Materials and Methods

### 4.1. Cell Culture and Treatment

The sensitive cell line DLD1 and the MDR cell line DLD1-TxR were cultured in Dulbecco′s Modified Eagle′s Medium (Capricorn Scientific, Ebsdorfergrund, Germany) supplemented with 10% fetal bovine serum (Capricorn Scientific) and 1% antibiotic/antimycotic solution (Capricorn Scientific) at 5% CO_2_ and 37 °C. Cells were subcultured with 1× trypsin/EDTA (Capricorn Scientific) after reaching 70–80% confluence. Cells were regularly examined for morphology using a microscope, while mycoplasma contamination was assessed using polymerase chain reaction (PCR). To cultivate cells in 3D, a scaffold-free method that relies on cells to self-assemble into spheroids was used.

Both cell lines were treated with two types of therapeutic agents: conventional chemotherapeutics (5-fluorouracil (EBEWE Pharma Ges.m.b.HNfg.KG, Unterach am Attersee, Austria), oxaliplatin (Actavis Italy S.p.A, Nerviano, Italy), and irinotecan (S.C. Sindan-Pharma S.R.L., Bucharest, Romania)) and biological agents (bevacizumab (F.Hoffmann-La Roche Ltd., Basel, Switzerland) and cetuximab (Merck KGaA, Darmstadt, Germany)). All therapeutic agents were freshly diluted in the medium to the final concentrations before use. For the treatment experiments, cells were trypsinized, washed twice with phosphate-buffered saline (PBS), and seeded at the appropriate density in different plates. Each experiment was performed in triplicate and included untreated control cells.

### 4.2. MTT Assay

The effects of all therapeutic agents on the viability of adherent cells were evaluated using the MTT assay. The assay was performed as a gold-standard colorimetric test to assess cell viability. Cells were seeded the day before treatment in a 96-well plate at a density of 2 × 10^3^. The next day, the medium was aspirated and replaced with fresh medium containing different concentrations of the conventional chemotherapeutic agents (10 nM, 100 nM, 1 µM, 10 µM, and 100 µM) and biological agents (50 µg/mL, 100 µg/mL, 250 µg/mL, 500 µg/mL, and 1000 µg/mL). The cells were incubated with the therapeutic agents for 72 h. The MTT assay was performed as previously described [80]. Cell viability was quantified by measuring absorbance at 550 nm on an automated Infinite 200 PRO multimode reader (Tecan Group Ltd., Männedorf, Switzerland) and data were analyzed using Magellan software (Tecan Group Ltd.). Cell viability was calculated using the following formula: Percentage of surviving cells (%) = (Absorbance value of sample/Absorbance value of control) × 100. The IC_50_ value represents the concentration of a cytotoxic drug that inhibits 50% of cell viability. The IC_50_ was calculated by nonlinear regression analysis in GraphPad Prism software v. 8.0.2 (GraphPad Software, LLC, San Diego, CA, USA). Rf, defined as the ratio of IC_50_ values between multidrug-resistant DLD1-TxR and drug-sensitive DLD1 cells (Rf = IC_50_[DLD1-TxR]/IC_50_[DLD1]), was used to assess the level of resistance.

### 4.3. Calcein-AM PI Viability Assay

Based on the results of the MTT test, the effects of the conventional chemotherapeutic agents (5-fluorouracil, oxaliplatin, and irinotecan) on the growth and viability of the spheroids were investigated using the ImageXpress^®^ Pico Automated Cell Imaging System (Molecular Devices, LLC, San Jose, CA, USA). For the formation of sensitive and resistant spheroids, DLD1 and DLD1-TxR cells were seeded at a density of 1.5 × 10^3^ in a Nunclon™ Sphera™ 96-well microplate with a U-shaped bottom (Thermo Fisher Scientific, Inc., Waltham, MA, USA), as previously described [81]. Cells were cultured into compact spheroids under the same conditions as adherent cells and treated with conventional chemotherapeutic agents at IC_50_ and 2 × IC_50_ concentrations for 72 h on the fourth day of cultivation.

To determine cell viability, sensitive and resistant spheroids were stained with calcein-AM (Tocris Bioscience, Bristol, UK) and PI (Sigma-Aldrich Chemie GmbH, Steinheim, Germany) to perform a live/dead assay. Calcein-AM labels viable cells with green fluorescence, while PI indicates non-viable cells by red fluorescence upon uptake. The spheroids were incubated for 15 min at 37 °C in medium containing 800 nM calcein-AM and 1 μM PI. After washing in 1 × PBS, the spheroids were imaged at 4× magnification, and Z-stack images were acquired. The projections of the Z-stack images were then used to quantify dead cells and spheroid size using ImageJ v. 1.52p software (U.S. National Institutes of Health, Bethesda, MD, USA). Quantification of dead cells was based on the mean fluorescence intensity of PI staining within the spheroids, while spheroid size was determined by area measurements.

### 4.4. Measurement of Chemotherapeutic Agents in the Cell Culture Medium

To determine the amount of chemotherapeutic agents in the medium, both cell lines were treated with conventional chemotherapeutic agents at their respective IC_50_ concentrations. The cells grown in 2D were seeded at a density of 2 × 10^5^ in 6-well plates and incubated overnight to allow them to attach to the plate surface. The next day, the cells were treated with the therapeutics at 37 °C for 4 h. After the incubation period, the medium was replaced with fresh medium, and the cells were left in the incubator to recover. After 2 h and 24 h of cell recovery, the cell culture medium was collected and analyzed by HPLC (Ultimate 3000, Thermo Fisher Scientific) on a 250 × 4.6 mm, 5.0 mm, Luna C18 (2) reversed-phase column (Phenomenex, Inc., Torrance, CA, USA). The compounds were separated at a flow rate of 1 mL min^−1^ using a mixture of acetonitrile and deionized water at 25 °C. The following elution procedure was used for the separation: 0–10 min, 100% water (isocratic step); 10–25 min, 100–80% water (linear gradient); 25–35 min, 80–60% water (linear gradient); 35–40 min, 60–100% water (linear gradient). Specific chemotherapeutic agents were identified by comparing the absorbance spectra with standards and by spiking. Quantification was based on the peak area with Chromeleon CDS 6.8 (Thermo Fisher Scientific) at 265 nm and 370 nm. LODs for irinotecan, 5-fluorouracil, and oxaliplatin were 5.62 nM, 1.56 nM, and 15.24 nM, respectively.

### 4.5. Nucleic Acid Isolation

Adherent DLD1 and DLD1-TxR cells were seeded at a density of 2 × 10^5^ in 24-well Nunclon™ Sphera™ plates (Thermo Fisher Scientific) with low adherence and cultured for 7 days to obtain spheroids. Untreated sensitive and resistant spheroids, without cell debris, were collected under a microscope for isolation of nucleic acids. According to the manufacturer’s instructions, DNA was isolated using the PureLink Genomic DNA Mini Kit (Thermo Fisher Scientific), while total RNA was isolated using the PureLink™ RNA Mini Kit (Thermo Fisher Scientific). The concentration and purity of the isolated nucleic acids were determined by absorbance at 260 nm and 280 nm using the BioSpec-nano spectrophotometer (Shimadzu Corporation, Kyoto, Japan). To minimize biological variability and ensure representativeness, nucleic acids isolated from three different plates were pooled for further sequencing.

### 4.6. Whole-Exome Sequencing

WES was performed by Novogene (UK) Company Limited (Cambridge, UK) using Illumina’s NovaSeq6000 platform. The WES workflow comprised of sample preparation (including DNA fragmentation into 180–280 bp fragments, which were end repaired, A-tailed, and further ligated with Illumina adapters), library preparation (including PCR amplification of fragments, their purification, and hybridization capture of WES libraries), and sequencing with 12 Gb (effective 50×) coverage. Paired-end clean reads were mapped to the human reference genome (hg38) using the Burrows–Wheeler aligner. The final bioinformatic analysis performed by Novogene resulted in a variant call format (.vcf file) containing insertion/deletions and single-nucleotide variants.

### 4.7. RNA-seq Analysis

High-throughput next-generation RNA-seq analysis, including bioinformatic analysis, was performed by Novogene (UK) Company Limited. Total RNA was quality-checked using 1% agarose gel electrophoresis, Nanodrop measurement to check RNA quantity and purity, and Agilent2100 measurement to determine RNA integrity number. Library preparation included depletion of ribosomal RNA, which enabled enrichment of RNA for gene expression profiling of coding transcripts. Paired-end sequencing (2 × 150 bp) was performed using Illumina’s NovaSeq6000 platform. Bioinformatic analysis included quality control (QC); removal of reads with adapters, reads with N > 0.1%, and low-quality reads (>50% bases showed QC ≤ 5); mapping of clean reads to the reference genome using HISAT2; and quantification of gene/transcript expression using Novogene’s well-established pipeline, resulting in the calculation of FPKM (Fragments Per Kilobase of transcript sequence per Millions base pairs) values for each transcript.

The Ballgown or cuffdiff tools were used to analyze the differential expression of genes, with a *p*-value < 0.05 considered statistically significant. Differentially expressed genes were visualized by a volcano plot, where the x-axis indicates the fold change in gene expression between different samples, and the y-axis indicates the statistical significance of the differences. GO enrichment analysis was performed for the differentially expressed genes, which were assigned to biological processes, molecular functions, and cellular components. The genes were assigned to GO terms, and the number of genes was calculated for each term. Subsequently, the Wallenius non-central hyper-geometric distribution was used to find significantly enriched GO terms in the genes compared to the background of the reference genes.

### 4.8. Statistical Analysis

Statistical analysis was performed using GraphPad Prism software v. 8.0.2 (GraphPad Software, LLC). Data were analyzed by one-way ANOVA, followed by Dunnett’s multiple comparisons test for comparisons between treatment groups and control and Tukey’s multiple comparisons test for pairwise comparisons between experimental groups. A significance level of *p* < 0.05 was accepted.

## 5. Conclusions

This study sheds light on the molecular mechanisms underlying MDR in colon cancer using the DLD1-TxR cell line model. Our results confirm significant resistance to conventional chemotherapeutic agents, especially oxaliplatin. The upregulation of genes related to ribosome biogenesis observed in resistant colonospheres suggests that increased ribosomal activity is a crucial mechanism for the acquisition of MDR. Targeting ribosome biogenesis together with specific deregulated genes may offer promising strategies to overcome MDR in colon cancer.

## Figures and Tables

**Table 1:** Half-maximal inhibitory concentration (IC_50_) values of therapeutics in DLD1 and DLD1-TxR cells.

	DLD1	DLD1-TxR	Rf ^a^
Chemotherapeutic agent, IC_50_ (µM)			
Paclitaxel ^b^	0.04	1.81	45
5-Fluorouracil	3.8	14.3	3.8
Oxaliplatin	0.12	124.8	1040
Irinotecan	1.9	2.7	1.4
Biological agent, IC_50_ (µg/mL)			
Bevacizumab	278.9	200	0.7
Cetuximab	>1000	>1000	n.d. *

^a^ Rf = IC_50_[DLD1-TxR]/IC_50_[DLD1]; ^b^ Podolski-Renic et al. [30]; * n.d., not-determined. Rf, relative resistance factor.

## Data Availability

Data are available from the corresponding author upon reasonable request.

## Supplementary Materials

The following supporting information can be downloaded at https://www.mdpi.com/article/10.3390/ijms26146580/s1.

## Abbreviations

The following abbreviations are used in this manuscript:

2D	Two-dimensional
3D	Three-dimensional
ANOVA	Analysis of variance
CRC	Colorectal cancer
DEGs	Differentially expressed genes
EGFR	Epidermal growth factor receptor
GO	Gene ontology
HPLC	High-performance liquid chromatography
IC_50_	The half-maximal inhibitory concentration
LOD	Limit of detection
MDR	Multidrug resistance
MRP1	MDR-associated protein 1
mTOR	Mammalian target of rapamycin
MTT	3-[4,5-dimethylthiazol-2-yl]-2,5-diphenyltetrazolium bromide
OCT2	Organic cation transporter 2
PBS	Phosphate-buffered saline
PCR	Polymerase chain reaction
P-gp	P-glycoprotein
PI	Propidium iodide
QC	Quality control
Rf	Relative resistance factor
RNA-seq	RNA sequencing
TCGA	The cancer genome atlas
WES	Whole-exome sequencing
