# Supplementary material for: Transcriptomic Landscape of Paclitaxel-Induced Multidrug Resistance in 3D Cultures of Colon Cancer Cell Line DLD1
Source: Int J Mol Sci. 2025 Jul 9;26(14):6580. doi: 10.3390/ijms26146580 (PMC12294787; doi:10.3390/ijms26146580)
Supplement: Supplementary file 1 [file ijms-26-06580-s001.zip › ijms-3642458-supplementary.pdf]

Transcriptomic Landscape of Paclitaxel-Induced Multidrug Resistance in 3D Cultures of Colon Cancer Cell Line DLD1

Sandra Dragicevic[1*], Jelena Dinic[2], Milena Ugrin[3], Marija Vidovic[4], Tamara Babic[1], Aleksandra Nikolic[1]

[1] Gene Regulation in Cancer Group, Institute of Molecular Genetics and Genetic Engineering, University of Belgrade, 11042 Belgrade, Serbia

[2] Department of Neurobiology, Institute for Biological Research "Siniša Stanković" - National Institute of the Republic of Serbia, University of Belgrade, 11108 Belgrade, Serbia

[3] Rare Disease Research and Therapeutics Development Group, Institute of Molecular Genetics and Genetic Engineering, University of Belgrade, 11042 Belgrade, Serbia

[4] Plant Molecular Biology Group, Institute of Molecular Genetics and Genetic Engineering, University of Belgrade, 11042 Belgrade, Serbia

(*) Corresponding author: Sandra Dragicevic**,** Gene Regulation in Cancer Group, Institute of Molecular Genetics and Genetic Engineering, University of Belgrade**,** Vojvode Stepe 444a, 11042 Belgrade, Serbia**,** E-mail: sandra.dragicevic@imgge.bg.ac.rs**;** ORCID ID: 0000-0002-1602-1880

Supplementary information

Table S1. Statistical comparison of drug responses (IC_{50} and $2\times IC_{50}$) between DLD1 and DLD1-TxR spheroids for oxaliplatin, irinotecan, and 5-fluorouracil

Table S2. Top upregulated genes in the DLD1-TxR versus DLD1 spheroids

Table S3. Top downregulated genes in the DLD1-TxR versus DLD1 spheroids

Figure S1. The sensitivity of parental DLD1 and multidrug resistant DLD1-TxR cells to chemotherapeutic agents

Table S1. Statistical comparison of drug responses (IC_{50} and $2\times IC_{50}$) between DLD1 and DLD1-TxR spheroids for oxaliplatin, irinotecan, and 5-fluorouracil.

**Treatment**	**Significance**	**Adjusted *p*-value**
Oxaliplatin IC_{50}	ns	>0.9999
Oxaliplatin $2\times IC_{50}$	ns	0.9111
Irinotecan IC_{50}	ns	>0.9999
Irinotecan $2\times IC_{50}$	ns	0.9999
5-fluoruracil IC_{50}	ns	>0.9999
5- fluoruracil $2\times IC_{50}$	ns	0.4738

Table summarizing one-way ANOVA Tukey's multiple comparisons test results between corresponding treatments in DLD1 and DLD1-TxR cell lines. The table only includes direct comparisons of the same treatment (IC_{50} or $2\times IC_{50}$) between DLD1 and DLD1-TxR.

Table S2. Top upregulated genes in the DLD1-TxR versus DLD1 spheroids.

Gene ID	Gene name	log2FoldChange	*p*-value	Adjusted *p*-value
ENSG00000162896	*PIGR*	13.2	1.57E-38	1.23E-34
ENSG00000261796	*ISY1-RAB43*	12.6	3.37E-34	1.36E-30
ENSG00000137674	*MMP20*	12.0	1.16E-29	2.74E-26
ENSG00000117148	*ACTL8*	11.0	1.97E-23	2.43E-20
ENSG00000284776	*AL121900.2*	10.5	6.87E-20	5.54E-17
ENSG00000016602	*CLCA4*	10.3	1.88E-18	1.21E-15
ENSG00000274600	*RIMBP3B*	10.2	4.79E-18	2.95E-15
ENSG00000178460	*MCMDC2*	9.7	2.65E-15	8.72E-13
ENSG00000125571	*IL37*	9.7	6.46E-15	2.03E-12
ENSG00000163286	*ALPPL2*	9.6	1.35E-14	3.99E-12
ENSG00000162897	*FCAMR*	9.6	1.63E-14	4.66E-12
ENSG00000263513	*FAM72C*	9.4	2.65E-13	5.61E-11
ENSG00000180447	*GAS1*	9.4	2.65E-13	5.61E-11
ENSG00000164266	*SPINK1*	9.3	7.79E-13	1.52E-10
ENSG00000143469	*SYT14*	9.2	1.91E-12	3.51E-10
ENSG00000117834	*SLC5A9*	9.1	6.14E-12	1.02E-09
ENSG00000249319	*AC068533.4*	8.8	9.92E-11	1.30E-08
ENSG00000267952	*AC008878.1*	8.6	6.95E-10	7.29E-08
ENSG00000284194	*SCO2*	8.6	6.95E-10	7.29E-08
ENSG00000117115	*PADI2*	8.6	9.30E-10	9.58E-08
ENSG00000174469	*CNTNAP2*	8.6	9.30E-10	9.58E-08
ENSG00000171403	*KRT9*	8.5	2.27E-09	2.09E-07
ENSG00000203778	*FAM229B*	8.5	2.27E-09	2.09E-07
ENSG00000276410	*HIST1H2BB*	8.4	7.89E-09	6.12E-07
ENSG00000128310	*GALR3*	8.4	7.89E-09	6.12E-07
ENSG00000106927	*AMBP*	8.3	1.51E-08	1.07E-06
ENSG00000179314	*WSCD1*	8.2	5.79E-08	3.45E-06
ENSG00000269113	*TRABD2B*	8.1	8.20E-08	4.65E-06
ENSG00000259823	*LYPD8*	8.1	8.20E-08	4.65E-06
ENSG00000142619	*PADI3*	8.1	1.17E-07	6.37E-06
ENSG00000109819	*PPARGC1A*	8.0	2.40E-07	1.19E-05
ENSG00000112562	*SMOC2*	7.9	5.06E-07	2.25E-05
ENSG00000274933	*TBC1D3I*	7.9	7.40E-07	3.14E-05
ENSG00000080493	*SLC4A4*	7.8	1.61E-06	6.19E-05
ENSG00000137965	*IFI44*	7.8	1.61E-06	6.19E-05
ENSG00000090382	*LYZ*	7.7	3.61E-06	0.000123
ENSG00000184716	*SERINC4*	7.6	5.45E-06	0.000175
ENSG00000162723	*SLAMF9*	7.6	5.45E-06	0.000175

ENSG00000158639	*PAGE5*	7.5	8.28E-06	0.00025
ENSG00000120949	*TNFRSF8*	7.5	8.28E-06	0.00025
ENSG00000166558	*SLC38A8*	7.5	8.28E-06	0.00025
ENSG00000183960	*KCNH8*	7.5	1.27E-05	0.000355
ENSG00000184005	*ST6GALNAC3*	7.5	1.27E-05	0.000355
ENSG00000204669	*C9orf57*	7.5	1.27E-05	0.000355
ENSG00000198074	*AKR1B10*	7.4	2.27E-27	4.29E-24
ENSG00000162344	*FGF19*	7.4	1.96E-05	0.000506
ENSG00000172318	*B3GALT1*	7.4	1.96E-05	0.000506
ENSG00000179954	*SSC5D*	7.4	1.96E-05	0.000506
ENSG00000213402	*PTPRCAP*	7.3	3.05E-05	0.000738
ENSG00000131910	*NR0B2*	7.3	3.05E-05	0.000738
ENSG00000198574	*SH2D1B*	7.3	3.05E-05	0.000738
ENSG00000170956	*CEACAM3*	7.3	4.78E-05	0.001075
ENSG00000019582	*CD74*	7.3	4.78E-05	0.001075
ENSG00000227507	*LTB*	7.3	4.78E-05	0.001075
ENSG00000130635	*COL5A1*	7.3	4.78E-05	0.001075
ENSG00000123892	*RAB38*	7.3	4.78E-05	0.001075
ENSG00000282246	*AL157392.5*	7.2	7.55E-05	0.001558
ENSG00000266265	*KLF14*	7.1	0.00012	0.002278
ENSG00000166736	*HTR3A*	7.1	0.00012	0.002278
ENSG00000196990	*FAM163B*	7.1	0.00012	0.002278
ENSG00000178568	*ERBB4*	7.1	0.00012	0.002278
ENSG00000262576	*PCDHGA4*	7.1	0.00012	0.002278
ENSG00000162511	*LAPTM5*	7.1	0.00012	0.002278
ENSG00000125999	*BPIFB1*	7.0	0.000194	0.003395
ENSG00000150636	*CCDC102B*	7.0	0.000194	0.003395
ENSG00000251246	*AL691442.1*	7.0	0.000194	0.003395
ENSG00000278272	*HIST1H3C*	7.0	0.000194	0.003395
ENSG00000100314	*CABP7*	7.0	0.000194	0.003395
ENSG00000180305	*WFDC10A*	7.0	0.000194	0.003395
ENSG00000086696	*HSD17B2*	7.0	0.000194	0.003395
ENSG00000142449	*FBN3*	7.0	0.000194	0.003395
ENSG00000070729	*CNGB1*	7.0	0.000194	0.003395
ENSG00000244255	*AL645922.1*	7.0	0.000194	0.003395
ENSG00000127588	*GNG13*	6.9	0.000315	0.005023
ENSG00000058091	*CDK14*	6.9	0.000315	0.005023
ENSG00000205143	*ARID3C*	6.9	0.000315	0.005023
ENSG00000104321	*TRPA1*	6.9	0.000315	0.005023
ENSG00000164659	*KIAA1324L*	6.8	0.000517	0.00734
ENSG00000249240	*AC069368.1*	6.8	0.000517	0.00734

ENSG00000107593	*PKD2L1*	6.8	0.000517	0.00734
ENSG00000126856	*PRDM7*	6.8	0.000517	0.00734
ENSG00000171916	*LGALS9C*	6.8	0.000517	0.00734
ENSG00000126970	*ZC4H2*	6.8	0.000517	0.00734
ENSG00000124233	*SEMG1*	6.8	0.000517	0.00734
ENSG00000212901	*KRTAP3-1*	6.8	0.000517	0.00734
ENSG00000105610	*KLF1*	6.8	0.000517	0.00734
ENSG00000129951	*PLPPR3*	6.7	0.000858	0.010955
ENSG00000163064	*EN1*	6.7	0.000858	0.010955
ENSG00000153976	*HS3ST3A1*	6.7	0.000858	0.010955
ENSG00000112769	*LAMA4*	6.7	0.000858	0.010955
ENSG00000285509	*AP000646.1*	6.7	0.000858	0.010955
ENSG00000135835	*KIAA1614*	6.7	0.000858	0.010955
ENSG00000174992	*ZG16*	6.7	0.000858	0.010955
ENSG00000170367	*CST5*	6.7	0.000858	0.010955
ENSG00000105246	*EBI3*	6.7	0.000858	0.010955
ENSG00000182901	*RGS7*	6.7	0.000858	0.010955
ENSG00000072182	*ASIC4*	6.6	3.54E-19	2.39E-16
ENSG00000007062	*PROM1*	6.6	1.22E-30	3.44E-27
ENSG00000130287	*NCAN*	6.5	0.001437	0.016307
ENSG00000172000	*ZNF556*	6.5	0.001437	0.016307
ENSG00000131152	*AC010531.1*	6.5	0.001437	0.016307
ENSG00000136931	*NR5A1*	6.5	0.001437	0.016307
ENSG00000173077	*DEC1*	6.5	0.001437	0.016307
ENSG00000109205	*ODAM*	6.5	0.001437	0.016307
ENSG00000182568	*SATB1*	6.5	1.11E-31	3.49E-28
ENSG00000221923	*ZNF880*	6.4	0.002435	0.024286
ENSG00000147082	*CCNB3*	6.4	0.002435	0.024286
ENSG00000113532	*ST8SIA4*	6.4	0.002435	0.024286
ENSG00000121410	*A1BG*	13.2	0.002435	0.024286
ENSG00000049768	*FOXP3*	12.6	0.002435	0.024286
ENSG00000090402	*SI*	12.0	0.002435	0.024286
ENSG00000156564	*LRFN2*	11.0	0.002435	0.024286
ENSG00000187957	*DNER*	10.5	0.002435	0.024286
ENSG00000182870	*GALNT9*	10.3	0.002435	0.024286
ENSG00000136688	*IL36G*	10.2	0.004174	0.036416
ENSG00000182771	*GRID1*	9.7	0.004174	0.036416
ENSG00000204882	*GPR20*	9.7	0.004174	0.036416
ENSG00000169248	*CXCL11*	9.6	0.004174	0.036416
ENSG00000159339	*PADI4*	9.6	0.004174	0.036416
ENSG00000184986	*TMEM121*	9.4	0.004174	0.036416

ENSG00000130643	*CALY*	9.4	0.004174	0.036416
ENSG00000165197	*VEGFD*	9.3	0.004174	0.036416
ENSG00000132938	*MTUS2*	9.2	0.004174	0.036416
ENSG00000174145	*NWD2*	9.1	0.004174	0.036416
ENSG00000140368	*PSTPIP1*	8.8	0.004174	0.036416
ENSG00000257529	*RPL36A-HNRNPH2*	8.6	2.62E-25	3.91E-22
ENSG00000007306	*CEACAM7*	8.6	1.23E-27	2.48E-24

Table S3. Top downregulated genes in the DLD1-TxR versus DLD1 spheroids.

Gene ID	Gene name	log2FoldChange	*p* value	Adjusted p value
ENSG00000124208	*TMEM189-UBE2V1*	-13.5	7.51E-41	1.06E-36
ENSG00000047648	*ARHGAP6*	-10.6	1.61E-20	1.58E-17
ENSG00000244115	*DNAJC25-GNG10*	-9.6	2.38E-14	6.49E-12
ENSG00000170486	*KRT72*	-8.8	1.30E-10	1.63E-08
ENSG00000166173	*LARP6*	-8.7	2.96E-10	3.36E-08
ENSG00000000971	*CFH*	-8.5	4.20E-09	3.59E-07
ENSG00000184995	*IFNE*	-8.4	7.89E-09	6.12E-07
ENSG00000173366	*AC097637.1*	-8.2	4.11E-08	2.59E-06
ENSG00000186377	*CYP4X1*	-8.2	5.79E-08	3.45E-06
ENSG00000204961	*PCDHA9*	-8.2	5.79E-08	3.45E-06
ENSG00000163959	*SLC51A*	-8.2	5.79E-08	3.45E-06
ENSG00000130948	*HSD17B3*	-8.1	1.17E-07	6.37E-06
ENSG00000174527	*MYO1H*	-7.9	5.06E-07	2.25E-05
ENSG00000166268	*MYRFL*	-7.9	7.40E-07	3.14E-05
ENSG00000091972	*CD200*	-7.8	1.09E-06	4.42E-05
ENSG00000121769	*FABP3*	-7.8	1.61E-06	6.19E-05
ENSG00000170465	*KRT6C*	-7.8	1.61E-06	6.19E-05
ENSG00000163462	*TRIM46*	-7.7	2.40E-06	8.62E-05
ENSG00000196724	*ZNF418*	-7.7	2.40E-06	8.62E-05
ENSG00000126233	*SLURP1*	-7.7	3.61E-06	0.000123
ENSG00000269026	*AC003006.1*	-7.6	5.45E-06	0.000175
ENSG00000116194	*ANGPTL1*	-7.6	5.45E-06	0.000175
ENSG00000131737	*KRT34*	-7.5	1.27E-05	0.000355
ENSG00000110665	*C11orf21*	-7.5	1.27E-05	0.000355
ENSG00000134760	*DSG1*	-7.5	1.27E-05	0.000355
ENSG00000105141	*CASP14*	-7.4	1.96E-05	0.000506
ENSG00000165935	*SMCO2*	-7.4	1.96E-05	0.000506
ENSG00000181541	*MAB21L2*	-7.4	1.96E-05	0.000506
ENSG00000137441	*FGFBP2*	-7.4	1.96E-05	0.000506
ENSG00000141316	*SPACA3*	-7.4	1.96E-05	0.000506
ENSG00000042980	*ADAM28*	-7.4	1.96E-05	0.000506
ENSG00000133574	*GIMAP4*	-7.3	3.05E-05	0.000738
ENSG00000056998	*GYG2*	-7.3	3.05E-05	0.000738
ENSG00000250673	*REELD1*	-7.2	4.78E-05	0.001075
ENSG00000260272	*AC093525.2*	-7.2	4.78E-05	0.001075
ENSG00000130433	*CACNG6*	-7.2	4.78E-05	0.001075
ENSG00000128591	*FLNC*	-7.2	7.55E-05	0.001558
ENSG00000197013	*ZNF429*	-7.2	7.55E-05	0.001558

ENSG00000157782	*CABP1*	-7.2	7.55E-05	0.001558
ENSG00000187959	*CPSF4L*	-7.2	7.55E-05	0.001558
ENSG00000241697	*TMEFF1*	-7.2	7.55E-05	0.001558
ENSG00000255432	*AP001458.2*	-7.1	0.00012	0.002278
ENSG00000129538	*RNASE1*	-7.1	0.00012	0.002278
ENSG00000129744	*ART1*	-7.1	0.00012	0.002278
ENSG00000240021	*TEX35*	-7.1	0.00012	0.002278
ENSG00000110900	*TSPAN11*	-7.1	0.00012	0.002278
ENSG00000284041	*AC073111.3*	-7.0	0.000315	0.005023
ENSG00000145536	*ADAMTS16*	-7.0	0.000315	0.005023
ENSG00000128383	*APOBEC3A*	-7.0	0.000315	0.005023
ENSG00000171051	*FPR1*	-7.0	0.000315	0.005023
ENSG00000189143	*CLDN4*	-7.0	1.74E-38	1.23E-34
ENSG00000253910	*PCDHGB2*	-6.9	0.000517	0.00734
ENSG00000244067	*GSTA2*	-6.9	0.000517	0.00734
ENSG00000005981	*ASB4*	-6.9	0.000517	0.00734
ENSG00000169760	*NLGN1*	-6.9	0.000517	0.00734
ENSG00000005961	*ITGA2B*	-6.9	0.000517	0.00734
ENSG00000169758	*TMEM266*	-6.9	0.000517	0.00734
ENSG00000145283	*SLC10A6*	-6.9	0.000517	0.00734
ENSG00000181322	*NME9*	-6.9	0.000517	0.00734
ENSG00000108950	*FAM20A*	-6.9	0.000517	0.00734
ENSG00000106571	*GLI3*	-6.9	0.000517	0.00734
ENSG00000233436	*BTBD18*	-6.8	0.000858	0.010955
ENSG00000240563	*L1TD1*	-6.8	0.000858	0.010955
ENSG00000163207	*IVL*	-6.8	0.000858	0.010955
ENSG00000128594	*LRRC4*	-6.8	0.000858	0.010955
ENSG00000117228	*GBP1*	-6.8	0.000858	0.010955
ENSG00000146122	*DAAM2*	-6.8	0.000858	0.010955
ENSG00000113578	*FGF1*	-6.8	0.000858	0.010955
ENSG00000212128	*TAS2R13*	-6.8	0.000858	0.010955
ENSG00000169413	*RNASE6*	-6.8	0.000858	0.010955
ENSG00000250254	*PTTG2*	-6.8	0.000858	0.010955
ENSG00000180525	*PRR26*	-6.8	0.000858	0.010955
ENSG00000127990	*SGCE*	-6.7	0.001437	0.016307
ENSG00000180549	*FUT7*	-6.7	0.001437	0.016307
ENSG00000152495	*CAMK4*	-6.7	0.001437	0.016307
ENSG00000091106	*NLRC4*	-6.7	0.001437	0.016307
ENSG00000244623	*OR2AE1*	-6.7	0.001437	0.016307
ENSG00000129596	*CDO1*	-6.7	0.001437	0.016307
ENSG00000108242	*CYP2C18*	-6.7	0.001437	0.016307

ENSG00000183287	*CCBE1*	-6.7	0.001437	0.016307
ENSG00000253159	*PCDHGA12*	-6.7	0.001437	0.016307
ENSG00000167751	*KLK2*	-6.7	0.001437	0.016307
ENSG00000234719	*NPIPB2*	-6.5	0.002435	0.024286
ENSG00000149256	*TENM4*	-6.5	0.002435	0.024286
ENSG00000187902	*SHISA7*	-6.5	0.002435	0.024286
ENSG00000244476	*ERVFRD-1*	-6.5	0.002435	0.024286
ENSG00000070915	*SLC12A3*	-6.5	0.002435	0.024286
ENSG00000145075	*CCDC39*	-6.5	0.002435	0.024286
ENSG00000152254	*G6PC2*	-6.5	0.002435	0.024286
ENSG00000110448	*CD5*	-6.4	0.004174	0.036416
ENSG00000101883	*RHOXF1*	-6.4	0.004174	0.036416
ENSG00000125363	*AMELX*	-6.4	0.004174	0.036416
ENSG00000138741	*TRPC3*	-6.4	0.004174	0.036416
ENSG00000171649	*ZIK1*	-6.4	0.004174	0.036416
ENSG00000178222	*RNF212*	-6.4	0.004174	0.036416
ENSG00000131233	*GJA9*	-6.4	0.004174	0.036416
ENSG00000103175	*WFDC1*	-6.4	0.004174	0.036416
ENSG00000148541	*FAM13C*	-6.4	0.004174	0.036416
ENSG00000081665	*ZNF506*	-6.4	0.004174	0.036416
ENSG00000156222	*SLC28A1*	-6.4	0.004174	0.036416
ENSG00000186912	*P2RY4*	-6.4	0.004174	0.036416
ENSG00000259075	*POC1B-GALNT4*	-6.3	4.71E-25	6.68E-22
ENSG00000135074	*ADAM19*	-5.9	3.39E-14	8.90E-12
ENSG00000256087	*ZNF432*	-5.9	1.97E-16	8.46E-14
ENSG00000126945	*HNRNPH2*	-5.9	1.11E-29	2.74E-26
ENSG00000139155	*SLCO1C1*	-5.9	8.36E-29	1.82E-25
ENSG00000197608	*ZNF841*	-5.9	2.48E-16	1.05E-13
ENSG00000196862	*RGPD4*	-5.8	8.64E-14	2.09E-11

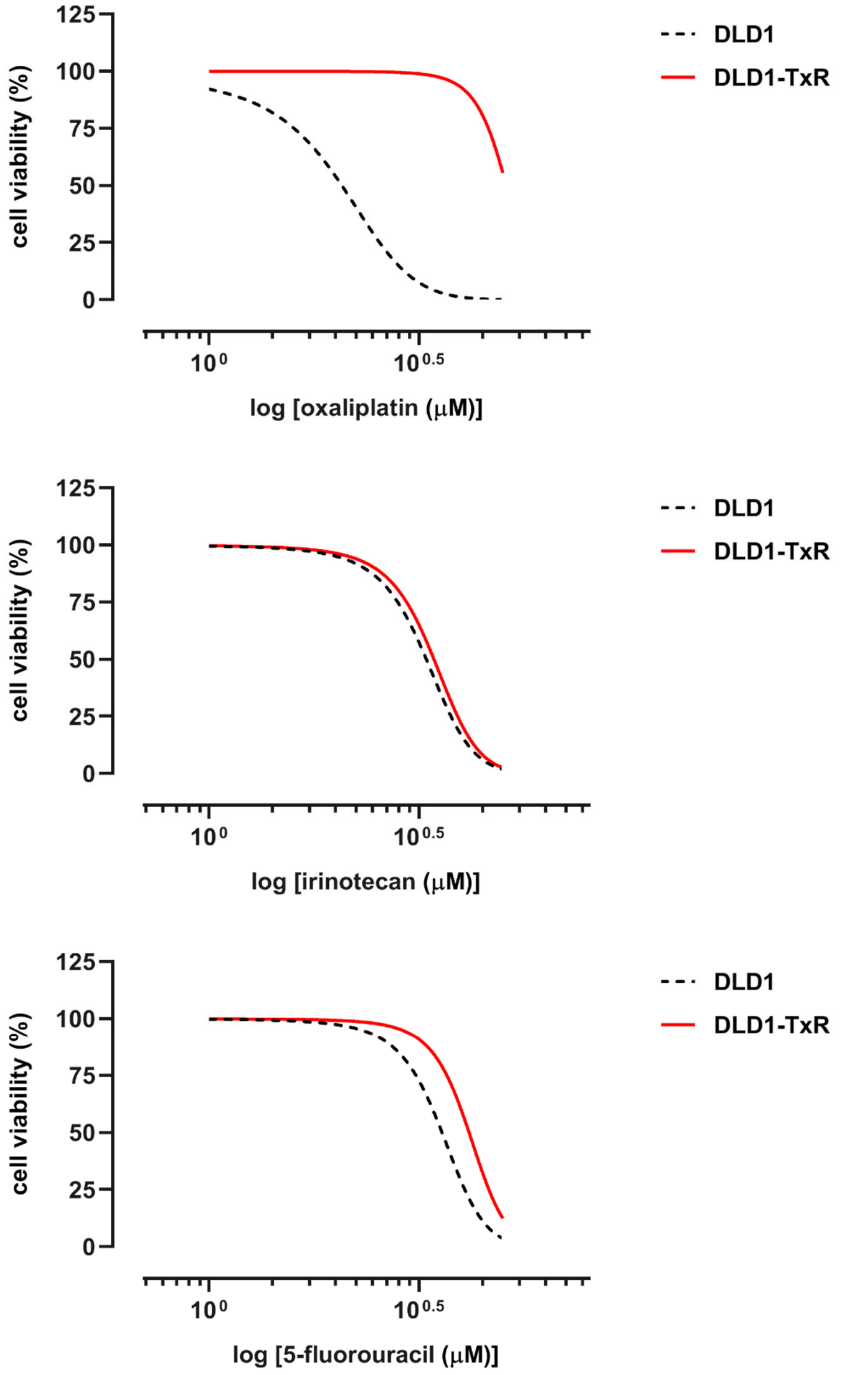

Figure S1. The sensitivity of parental DLD1 and multidrug resistant DLD1-TxR cells to chemotherapeutic agents. The effects of oxaliplatin, irinotecan and 5-fluorouracil, on cell growth inhibition were assessed by MTT assay after 72 h. The graphs represent nonlinear regression fitted curves generated by GraphPad Prism software version 8.0.2 (GraphPad Software, LLC, San Diego, CA, USA).
